# Recurrence of Non-Hydropic Sudden Sensorineural Hearing Loss (SSNHL): A Literature Review

**Published:** 2019-01-12

**Authors:** C Cassandro, P De Luca, M Ralli, F Gioacchini, F Di Berardino, A Albera, R Albera, E Cassandro, A Scarpa

**Affiliations:** 1 Surgical Sciences Department, University of Turin, Turin, Italy; 2 Department of Medicine and Surgery, University of Salerno, Salerno, Italy; 3 Department of Sense Organs, Sapienza University Rome, Rome, Italy; Center for Hearing and Deafness, University at Buffalo, Buffalo, NY 14214, USA; 4 Ear, Nose, and Throat Unit, Department of Clinical and Molecular Sciences, Polytechnic University of Marche, Ancona, Italy; 5 Audiology Unit, Department of Clinical Sciences and Community, Fondazione IRCCS Ca’ Granda Ospedale Maggiore Policlinico, Università degli Studi di Milano, Milan, Italy

## 1. Introduction

Sudden Sensorineural Hearing Loss (SSNHL) is typically defined as the acute onset (less than 3 days) of a perceptive hearing loss of more than 30dB over at least three contiguous frequencies on pure tone audiometry [1]. The exact incidence of SSNHL is uncertain, since many patients have a rapid and spontaneous resolution of symptoms and therefore don’t reach medical attention. Estimate of incidence ranges from 5 to 20 per 100.000 individuals, and bilateral involvement is very rare [2]; it increases in the older patients (>65 yo)(77 per 100.000) the in younger population (<18 yo)(11 per 100.000) [3]. The true incidence of paediatric SSNHL is not established in literature; 40% of examined child with SSNHL, showed anatomic abnormalities [4].

The left ear is more affected only in female patients; the cause is unclear, but this asymmetry might indicate a greater vulnerability only in female, due to hormonal factors in the genesis of sudden deafness [5].

**Figure 1:**
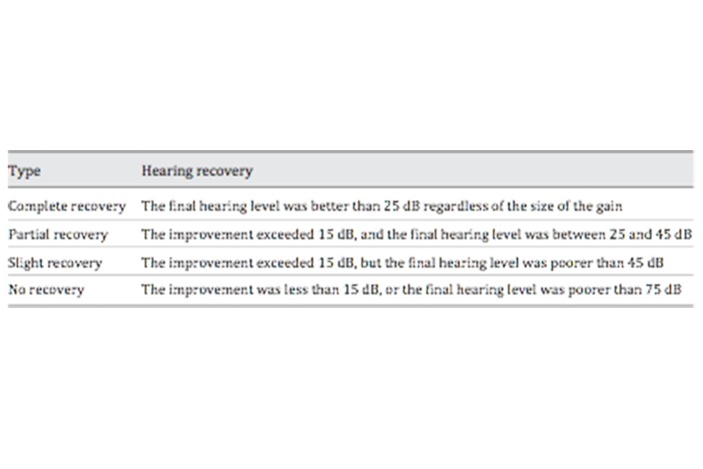
Cause of Sudden Sensorineural Hearing Loss (from “Hearing loss in adults: differential diagnosis and treatment”, Michels TC et al., Am Fam Physician 2019 Jul 15;100(2):98–108)

The pathogenesis of SSNHL it has been widely studied but is not yet entirely clear: it is multifactorial, and various causes have been proposed, including viral infection of the labyrinth or cochlear nerve, autoimmune ear disease, acoustic tumors, perilymphatic fistula, intracochlear membrane trauma or rupture, drug toxicity, vascular disorders (including hemorrhaging, thrombosis, embolism, vasospasm and hypercoagulability in the microcirculation of the cochlea)[6], Meniere’s disease [7] **(**Figure 1); in addition, major depression ad anxiety can be the cause of sudden deafness [8].

However, in most patients the cause is hidiopatic.

It is commonly presented as a sudden unilateral deafness on awakening; aural fullness, tinnitus, vertigo and dysequilibrium are present to a variable degree in approximately 40% of patients [9].

The clinician should exclude a conductive haring loss, perform an audiometry test as soon as possibile (within 14 days of symptoms onset) and evaluate the patient for retrocochlear pathology by optioning an MRI or auditory brainstem response (ABR); CT scan ad routine laboratory tests are not recommended in the approach.

**Figure 2:**
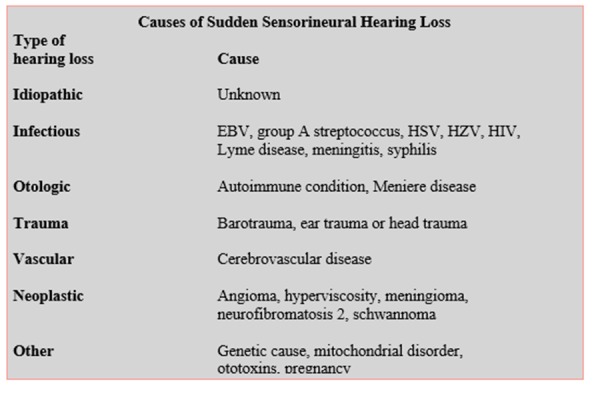
Siegel’s criteria: degree of hearing recovery from sudden hearing loss

Most recovery occurs within the first 2 weeks after onset; without treatment of any kind, a significant proportion (30% to 65%) of patients experience complete or partial recovery. (Figure 2).

Children and adults >40 years old have a poorer prognosis [2,10].

Treatment of SSNHL should be based on its etiology. Most cases are idiopathic; the best options is initial therapy with corticosteroids, which can be combined with hyperbaric oxygen therapy within 2 weeks of onset of SSNHL.

Hyperbaric oxygen therapy (HBO) can be offered or combined with steroid therapy within 2 weeks of onset of SSHl, or combined with steroid therapy as salvage within 1 month of onset of symptoms. Some authors [11] recommend the routine application of HBO with intravenous steroid for all patients with idiopathic sudden sensorineural hearing loss within 14 days from the onset of symptoms.

Antivirals, trombolytics and a vasodilatators are not recommended.

When patients have incomplete recovery 2 to 6 weeks after onset of symptoms, intratympanic steroids could be an option as salvage therapy [1].

Meniere syndrome can occur in 5% of cases with SSNHL; generally, other symptoms are present (vertigo, tinnitus)[12,13]. Schaaf et al. [14] reported that recurrent low-frequency sensory hearing loss can be one characteristic of Meniere’s disease; in this work, only 3.7% of the patients developed the typical signs of Meniere’s disease, and 25.9% of these suffered from recurrent hearing loss. They conclude that although almost every patient with Meniere’s disease suffers from recurrent hearing loss, only a few patients with recurrent hearing loss develop Meniere’s disease.

Acute low-tone sensorineural hearing loss (ALSHL) is a deterioration in audiometric low frequencies (250–500 Hz) with preservation of high frequencies; it was initially considered as a variant of SSNHL, until it was described as an independent disease entity. Moreover, there are several differences beetween ALSHL and SSNHL; 1) predominance of female (72.9%), 2) more dizzines (36%) and tinnitus (42.8%), 3) ear fullness (20.8%), 4)better prognosis of complete recovery (from 67.7% to 77%), 5) absence of predictive factors, 6) an important develop to Meniere’s disease 15%) [15].

Additionally, the incidence of relapse is higher than in SSNHL (24%) [16,17].

Although there are many reports about the pathogenesis, treatment and prognostic criteria of SSNHL, few studies have examined relapsed neurosensorial hearing loss.; moreover, most of the studies do not investigate the pathologies leading to relapses.

The aim of this paper is to evaluate the incidence and the risk factors of recurrent non-hydropic SSNHL in literature.

## 2. Discussion

We define SSNHL relapse as an episode of SSNHL greater than or equal than the first episode of SSNHL; it consists in two types, ipsilateral and controlateral SSNHL; the ipsilateral type is defined as the first episode of idiopathic sudden deafness that had hearing improvement; after a period, a second episode of sudden deafness recurred in the same ear. In the contralateral type, two episodes of sudden deafness occurred in each ear, alternatively [18]. Kuo YL et al. comparised the two types, and no significant differences existed in term of 1) age of onset at the second episode, 2) gender, 3) laterality, 4) presence of vertigo, 5) spontaneous nystagmus, 6) abnormal ENG findings, 7) abnormal caloric results, 8) initial and final mean hearing. A significant difference was observed in abnormal VEMPs between the two types; all patients with ipsilateral type had normal VEMPs associated with improved hearing, whereas all patients with contralateral type showed abnormal VEMPs. In conclusion, the demonstration of normal VEMPs in the lesion ear of the second wayside in patients with recurrent SSNHL may indicate a good hearing outcome.

The main cause (90%) of SSNHL is idiopathic sudden sensorineural hearing loss (ISSNHL) in which the cause is not identifiable (o undetermined); is commonly unilateral, and bilateral loss should lead thinking about other causes. It is not commonly associated with other symptoms.

The incidence of ISSNHL relapse reported in the English Literature varie from 0.8% [19] to 8.3% [20].

The definite pathogenesis of ISSNHL is currently unclear

Che-Ming Wu et. al [21] described one of the largest cohort of patients with ISSNHL; a relapse od ISSNHL occurred in 2.281 patients (incidence 4.99%). The relapse was significantly higher (60%) in middle age patients (35–64 yo) than in younger patients (0–34 yo) (17%). In this study there is an higher prevalence of comorbid diabetes mellitus and hypercholesterolemia in the patients with a recurrence of ISSNHL than in those without, suggesting that the insufficient cochlear perfusion is an important risk factor for the recurrence of sudden deafness; otherwise, the individual comorbidities of coronary artery disesase, hypertension, chronic renal disease, diabetes mellitus and hypercholesterolemia are not associates with an higher incidence of ISSNHL. There is no a large difference in relapse between male and female (54.11% vs. 45.89%).

Identifiable causes are found for 7% to 45% of patients with SSNHL [22, 23, 24].

The ethiology is very broad; Chau et al. [25], identified infections (13%), otologic problems (5%), traumatic (4%), vascular (3%), neoplastic (2%) as the most frequent cause of symptomatic sudden hearing loss. Capuano et al [26] suggested that patent forman ovale and right to left shunt are frequent in ISSNHL, particularly in young patients without comorbidities and with associated dizziness.

Infectious, traumatic, metabolic and neurologic disease are not linked to relapsed SSNHL (although increased levels of neutrophil to lymphocyte ratio (NLR) e platelet to lymphocyte ratio (PLR) have been shown in relapse SSNHL [27]. About infections in recurrent SSNHL, some authors [28] speculate that are caused by a reactivation of a latent HSV infection; in some patient, high values of serum antibody titer for adenovirus 3 or human HSV-1 or VZV were detected.

Some vascular diseases can cause recurrent SSNHL, usually associated with other symptoms.

Lee and Cho [29] proposed some auditory disturbance (as recurrent sudden sensorineural hearing loss) as a warning sing of impeding pontocerebellar infarction in the distribution of anterior inferior cerebellar artery (AICA) due to the ischemia of the inner ear or the vestibulocochlear nerve. In the opinion of Park JH et al. [30], in elderly patients (expecially with particular risk factors) with recurrence of hearing loss and vertigo lasting several minutes, physicians may also consider the potential symptom of AICA infarction. Park H et. al [31] reported a single case of unilateral recurrent hearing loss due to medullary infarction.

Bliss et al. [32] described an interesting report about recurrent contralateral hearing loss after two craniotomies for vestibular schwannoma; SSNHL on the the side opposite cerebellopontine angle surgery is a rare but well-documented occurrence. Most losses are recurrent but transient; the cause for the contralateral loss is still unclear; auto-immune mediated cocheolabyrinthitis [33], cardiovascular complication [34], allergy [35], labyrinthine rupture from elevated intratympanic pressure during general anestethic [36] are just theories.

Some authors, as Walsted et al. [37] proposed the concept of a relative endolymphatic hydrops in the controlateral ear after vestibular schwannoma removed; they suggested that low CSF pressure can be transmitted to the cochlea through the cochlear aqueduct, creating a low pelymphatic pressure.

Some neoplasm may be related to recurrent SSNHL; for example, a vestibular schwannoma or another tumor in the internal auditory canal or at the cerebellopontine angle should be the cause [7].

MRI imaging should be performed to evaluate a vascular or retrocochlear pathology.

Electrocochleographic findgs should be assessed as prognostic factors in ISSNHL; in a study by Ohashi et al. [38], the patients were examined with transtympanic trasnpromontory ECochG within 5 days from the onset of symptoms; they concluded that an enhanced SP/AP ratio and a low initial AP threshold may be positive prognostic factors in recurrent ISSNHL; furthermore the presence of initial vertigo seems to be an unfavourable factor in recurrent ISSNHL.

Prognosis and recurrence rate can be influenced by many factors; metabolic syndrome [39], hyperlipidemia [40], diabetes mellitus [41,42,43,44,45] are negative prognostic factors for recovery and recurrences; furthermore, hearing recovery is poorer after a second attack of SSNHL.

## 3. Conclusion

Although recurrent non-hydropic SSNHL is unfrequent, it represents a diagnostic and therapeutic challenge.

Differential diagnosis is essential to distinguish idiopathic forms from those symptomatic: the clinician should pay attention to vascular disease and retrocochlear neoplasm.

The literature established that recurrent SSNHL has a poorer prognosis than a single episode of sudden hearing loss.

Although some factors are described to affect recovery and recurrent state, further studies should needed to explain better the related mechanisms and to evaluate the role of audiometric examinations.

## References

[1] Chandrasekhar S (2019). Clinical Practice Guideline: Sudden Hearing Loss (Update). Otolaryngol Head Neck Surg.

[2] Bye FM (1984). Sudden Hearing Loss: Eight Year’s Experience and suggested prognostic table. Laryngoscope.

[3] Alexander TH (2013). Incidence of sudden sensorineural hearing loss. Otol Neurotol.

[4] Dedhia K (2016). Pediatric sudden sensorineural hearing loss: etiology, diagnosis and treatment in 20 children. Int J Pediatr Otorhinolaryngol.

[5] Reiss M (2014). Laterality of sudden sensorineural hearing loss. Ear Nose Throat J.

[6] Capaccio P (2007). Genetic and acquired prothrombotic risk factor and sudden hearing loss. Laryngoscope.

[7] Park IS (2013). Clinical Analysis of Recurrent Sudden Sensorineural Hearing Loss. ORL J Otorhinolaryngol Relat Spec.

[8] Mori S (2002). Psycogenic Hearing Loss With Panic Attack After The Onset Of Acute Inner Disorder. ORL J Otorhinolaryngol Relat Spec.

[9] Mattox DE (1977). Natural History of Sudden Sensorineural Hearing Loss. Ann Otol Rhinol Laringol.

[10] Huafeng Y (2019). Clinical characteristics and prognosis of elderly patients with idiopathic sudden sensorineural hearing loss. Acta Otolaryngol.

[11] Capuano (2015). Hyperbaric oxygen for idiopathic sudden hearing loss: is the routine application helpful?. Acta Otolaryngol.

[12] Scarpa A (2019). Clinical application of cVEMPs and oVEMPs in patients affected by Meniere’s disease, vestibular neuritis and benign paroxysmal positional vertigo: a systematic review. Acta Otorhinlaryngol Ital.

[13] Scarpa A (2019). Low dose intratympanic administration for unilateral Meniere’s disease using a method based on clinical symptomatology: preliminary results. Am J Otolaryngol.

[14] Schaaf (2001). Is recurrent loss of low frequency tone perception—without vertigo—a precursor of Meniere disease?. HNO.

[15] Psilla (2019). Hearing outcome of low-tone compared to high-tone sudden sensorineural hearing loss. International Archives of Othorinolaryngology.

[16] Yoshida T (2017). Idiopathic sudden sensorineural hearing loss and acute low-tone sensorineural hearing loss: a comparison of the results of a nationwide epidemiologica survey in Japan. Acta Otolaryngologica.

[17] Scarpa A (2019). Food-induced stimulation of the anti secretory factor to improve symptoms in Meniere’s disease: our results. Eur Arch Othorinolaryngol.

[18] Kuo YL (2012). Hearing outcome of recurrent sudden deafness: ipsilateral versus contralateral types. Acta Otolaryngol.

[19] Furuashi A (2002). Sudden deafness: long term follow-up and recurrence. Clinic Otolaryngol.

[20] Eichorn T (1984). Course and prognosis in sudden deafness. HNO.

[21] Wu Che-Ming (2014). Recurrence of Idiopathic Sudden Sensorineural Hearing Loss: a Retrospective Cohort Study. Otol & Neurotol.

[22] Byl FM (1984). Sudden hearing loss: eight years’ experience and suggested prognostic table. Laryngoscope.

[23] Nosrati (2007). Idiopathic sudden sensorineural hearing loss: results drawn from the Swedish national database. Acta Otolaryngol.

[24] Huy PT (2005). Idiopathic sudden sensorineural hearing loss is not an otologic emergency. Otol Neurotol.

[25] Chau JK (2010). Systematic review of the evidence for the etiology of adult sudden sensorineural hearing loss. Laryngoscope.

[26] Capuano L (2019). Right-to-left shunt and idiopathic sudden sensorineural hearing loss, Capuano L et al. Acta Otorhinolaryngol Ital.

[27] Set YJ (2015). Predictive valute of neutrophil to lymphocyte ratio in first-time and recurrent idiopathic sudden sensorineural hearing loss. Auris Nasus Larynx.

[28] Ohashi (2013). Electrocochleographic findings in recurrent idiopathic sudden sensorineural hearing loss. Otol & Neurotol.

[29] Lee H (2003). Auditory disturbance as prodrome of anterior inferior cerebellar artery infarction. J Neurol Neurosurg Psychiatry.

[30] Park JH (2008). Recurrent audiovestibular disturbance initially mimicking Meniere’s disease in a patient with anterior inferior cerebellar infarction. Neurol Sci.

[31] Park HJ (2018). A case of medullary infraction presented initial symptoms similar to Meniere’s disease. J Audiol Otol.

[32] Bliss MR (2013). Recurrent contralateral hearing loss after 2 craniotomies for vestibular schwannoma: etiologic implications. Otol & Neurotol.

[33] Harris JP (1985). Contralateral hearing loss following inner ear injury: sympathetic cochleolabyrinthitis?. Am J Otol.

[34] Millen SJ (1982). Sudden sensorineural hearing loss: operative complication in non-otologic surgery. Laryngoscope.

[35] Clemis JD (1982). Sudden hearing loss in the contralateral ear in postoperative acoustic tutor: three case reports. Laryngoscope.

[36] Segal S (1984). Labyrinthine membrane rupture caused by elevated intratympanic pressure during general anaesthesia. Am J Otol.

[37] Walsted A (1991). Hearing decrease after loss of cerebrospinal fluid. A new hydrops model?. Acta Otolaryngol.

[38] Ohashi (2012). Electrocochleographic findings in recurrent idiopathic sudden sensorineural hearing loss. Acta Otolaryngol.

[39] Zhang Y (2019). The influence of metabolic syndrome on the prognosis of idiopathic sudden sensorineural hearing loss. Otol Neurotol.

[40] Chen C (2019). Impact of hyperlipidemia as a coexisting factor on the prognosis of idiopathic sudden sensorineural hearing loss: a propensity score matching analysis. Clin Otolaryngol.

[41] Stolzel K (2018). Comorbid symptoms occurring during acute low-tone hearing loss as potential predictors of Meniere’s disease. Front Neurol.

[42] Gioacchini FM (2018). Hyperglycemia and diabetes mellitus are related to vestibular organs dysfunction: truth or suggestion? A literature review. Acta Diabetol.

[43] Gioacchini FM (2019). The role of diabetes mellitus in favouring peripheral vestibular system dysfunctions: clinical and scientific evidence. Otorinolaringol.

[44] Moschen R (2019). Validation of the chronic tinnitus acceptance questionnaire 8CTAQ-I): the Italian version. Acta Otorhinolaryngol Ital.

[45] Cassandro E (2015). Inner ear conductive hearing loss and unilateral pulsatile tinnitus associated with a dural arteriovenous fistula: case based review and analysis of relationship between intracranial vascular abnormalities and inner ear fluids. Case Rep Otolaryngol.

